# Interreader and Intrareader Reproducibility of ^18^F-Flotufolastat Image Interpretation in Patients with Newly Diagnosed or Recurrent Prostate Cancer: Data from Two Phase 3 Prospective Multicenter Studies

**DOI:** 10.2967/jnumed.123.267306

**Published:** 2024-08

**Authors:** Phillip H. Kuo, Giuseppe Esposito, Gary A. Ulaner, Don Yoo, Katherine Zukotynski, Gregory C. Ravizzini, Ross Penny, Matthew P. Miller, Albert Chau, Phillip Davis, Brian F. Chapin, David M. Schuster, on behalf of the SPOTLIGHT and LIGHTHOUSE study groups

**Affiliations:** 1 Departments of Medical Imaging, Medicine, and Biomedical Engineering, University of Arizona, Tucson, Arizona;; 2 Department of Nuclear Medicine, Medstar Georgetown University Hospital, Washington, District of Columbia;; 3 Department of Molecular Imaging and Therapy, Hoag Family Cancer Institute, Irvine, California;; 4 Radiology and Translational Genomics, University of Southern California, Los Angeles, California;; 5 Warren Alpert Medical School of Brown University, Providence, Rhode Island;; 6 Department of Radiology, McMaster University, Hamilton, Ontario, Canada;; 7 Division of Diagnostic Imaging, Department of Nuclear Medicine, University of Texas MD Anderson Cancer Center, Houston, Texas;; 8 Blue Earth Diagnostics Ltd., Oxford, United Kingdom;; 9 Blue Earth Diagnostics Inc., Monroe Township, New Jersey;; 10 Department of Urology, University of Texas MD Anderson Cancer Center, Houston, Texas; and; 11 Division of Nuclear Medicine and Molecular Imaging, Department of Radiology and Imaging Sciences, Emory University, Atlanta, Georgia

**Keywords:** molecular imaging, oncology, PET/CT, PSMA, interreader variability

## Abstract

Interreader and intrareader reproducibility of ^18^F-flotufolastat PET/CT scans in newly diagnosed and recurrent prostate cancer patients was assessed from masked image evaluations from two phase 3 studies. **Methods:** ^18^F-flotufolastat PET/CT images of newly diagnosed (*n* = 352) or recurrent (*n* = 389) patients were evaluated by 3 masked readers. Cohen κ was used to assess pairwise patient- and region-level interreader agreement. Agreement among all readers was assessed using Fleiss κ. Intrareader agreement between the first and repeat read (20% of images, ≥4 wk later) was assessed using Cohen κ. **Results:** Pairwise interreader agreement was 95% or better (newly diagnosed) and 75% or better (recurrent). The κ coefficients were impacted by the high-agreement–low-κ paradox: Cohen κ ranged from not estimable to 0.55, whereas Fleiss κ was 0.50 (newly diagnosed) and 0.41 (recurrent). Agreement was highest in the prostate of newly diagnosed patients (≥95%) and in the pelvic lymph nodes in recurrent patients (≥87%). Intrareader agreement was 86% or better across both populations. **Conclusion:** ^18^F-flotufolastat PET/CT images can be reliably interpreted, with a high degree of inter- and intrareader agreement.

## Visual Abstract

**Figure:**
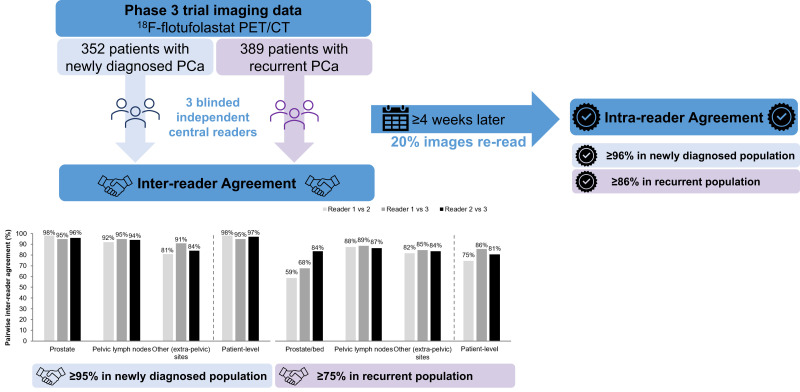

Prostate-specific membrane antigen (PSMA) PET has become a standard of care for prostate cancer (PCa) imaging, as demonstrated by its inclusion in the most recent guidelines from the National Comprehensive Cancer Network (1).

^18^F-flotufolastat is a high-affinity PSMA-targeting PET diagnostic radiopharmaceutical developed from a radiohybrid technology platform that enables engineering of PSMA ligands that can be labeled with ^18^F for diagnostic imaging or with α- or β-emitting radiometals for radiopharmaceutical therapy (2). The data from two phase 3 clinical studies, LIGHTHOUSE (NCT04186819) and SPOTLIGHT (NCT04186845), show ^18^F-flotufolastat to be well tolerated and to provide clinically useful information regarding the presence of N1 and M1 disease before surgery in newly diagnosed PCa patients (3) and for localization of recurrent PCa (4). Data from the SPOTLIGHT study showed the patient-level verified detection rate of ^18^F-flotufolastat in patients with recurrent prostate cancer to be 51%–54% (4), whereas data from the LIGHTHOUSE study showed that in newly diagnosed prostate cancer, ^18^F-flotufolastat had a sensitivity of 23%–30% and a specificity of 93%–97% for the detection of pelvic lymph node (PLN) metastases (3). ^18^F-flotufolastat was recently approved by the U.S. Food and Drug Administration for diagnostic PET imaging of PSMA-positive lesions in men with PCa (5) and was included in the most recent guideline updates from National Comprehensive Cancer Network and the American Society of Clinical Oncology (1*,*^6^).

Here, we evaluate the results of masked image evaluations from the LIGHTHOUSE and SPOTLIGHT studies to investigate whether masked readers could, after training, reliably interpret ^18^F-flotufolastat PET/CT images in the newly diagnosed or recurrent setting. We report the interreader and intrareader reproducibility of ^18^F-flotufolastat for each population.

## MATERIALS AND METHODS

The full methods of the LIGHTHOUSE and SPOTLIGHT studies have been reported previously (3*,*^4^). In brief, treatment-naïve men aged older than 18 y with biopsy-proven PCa and unfavorable intermediate-risk to high-risk disease classification who were scheduled for radical prostatectomy with regional PLN dissection were enrolled in the LIGHTHOUSE study. The SPOTLIGHT study recruited men aged older than 18 y with elevated prostate-specific antigen (PSA) suspected to be biochemical recurrence after curative-intent treatment of localized PCa if they were eligible for curative-intent salvage therapy. Patients in both studies received 296 MBq (8 mCi ± 20%) of ^18^F-flotufolastat, and PET/CT imaging was conducted 50–70 min after injection. Each site used either a current- or previous-generation dedicated PET/CT system, with time-of-flight capabilities. All systems were approved by the imaging core lab before patients were scanned.

In both studies, all scans were evaluated by 3 masked central readers. Two individuals were readers on both studies. The readers were all board-certified nuclear medicine physicians with more than 15 y of experience who underwent protocol-specific, ^18^F-flotufolastat–specific training that included marked assessment cases to ensure alignment with an expert reader. The readers were masked to all clinical information. The readers were instructed that lesions should be considered suggestive if ^18^F-flotufolastat uptake was greater than physiologic uptake in that tissue or greater than the adjacent background if no physiologic uptake was expected (3*,*^4^).

### Pairwise Inter- and Intrareader Agreement

Pairwise inter- and intrareader agreements were assessed as secondary endpoints in both studies and were reported at a patient level and for the prostate and prostate bed, PLN, and other (extrapelvic) regions (i.e., extra-PLN, soft tissue or parenchyma, and bone). The Cohen κ statistic (7) was used to test pairwise agreement between any 2 readers, giving 3 κ statistics. To assess intrareader agreement, each reader conducted a reread of a random selection of 20% of the images, with the repeat read taking place at least 4 wk after the first read. Cohen κ was used to assess agreement between the 2 reads.

### Overall Interreader Agreement Among All 3 Readers

The patient-level agreement among all 3 readers was performed as a post hoc assessment during regulatory review using the Fleiss κ statistic (8). For this metric, agreement on lesions in 5 distinct regions (prostate or prostate bed, PLN, extra-PLN, soft tissue or parenchyma, and bone) was considered part of the assessment.

### Statistical Analyses

All κ coefficients were calculated using SAS (version 9.4; SAS Institute) and are presented with approximate 95% CIs.

## RESULTS

### Patients

In total, 352 scans from patients in the LIGHTHOUSE study and 389 scans from patients in the SPOTLIGHT study were assessed by the readers to determine the inter- and intrareader agreement. The 352 patients in the LIGHTHOUSE study with newly diagnosed PCa had a median age of 65 y and a median PSA of 8.8 ng/mL, and 32% of those patients were considered to have unfavorable intermediate-risk disease. The 389 patients in the SPOTLIGHT study with recurrent PCa had a median age of 69 y and a median PSA of 1.1 ng/mL, and 79% of those patients had previously undergone radical prostatectomy.

### ^18^F-Flotufolastat–Positive Lesions

In the LIGHTHOUSE study, across the 3 readers, 335–352 (95%–100%) of the 352 patients with newly diagnosed PCa had ^18^F-flotufolastat–positive lesions. On a regional basis, 333–352 (95%–100%) patients were positive in the prostate, 44–61 (13%–17%) patients were positive in PLN, and 56–98 (16%–28%) patients were positive in extrapelvic sites.

In the SPOTLIGHT study, across the 3 readers, 264–358 (68%–92%) of the 389 patients with recurrent PCa had ^18^F-flotufolastat–positive lesions. In total, 101–256 (26%–66%) patients were positive in the prostate region, 111–138 (29%–36%) patients were positive in PLN, and 146–173 (38%–44%) patients were positive in extrapelvic sites.

### Interreader Agreement

**FIGURE 1.**
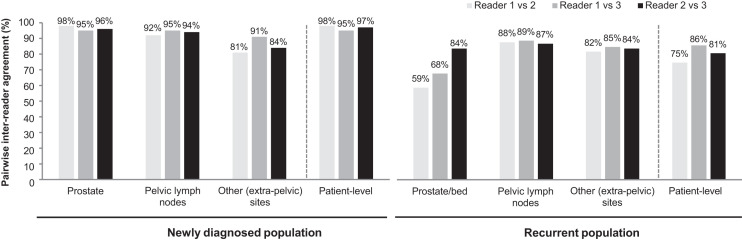
Pairwise patient- and region-level interreader agreement for 352 newly diagnosed and 389 recurrent PCa patients with evaluable ^18^F-flotufolastat PET/CT scan.

Patient-level, pairwise interreader agreement comparisons were 95% or better in the newly diagnosed population and 75% or better in the recurrent population (Fig. 1). On a regional basis, the pairwise agreement was greater than 80% for all regions except the prostate or prostate bed in the recurrent population. The pairwise agreement was highest in the prostate among the newly diagnosed population and highest in PLN in the recurrent population (Fig. 1).

**FIGURE 2.**
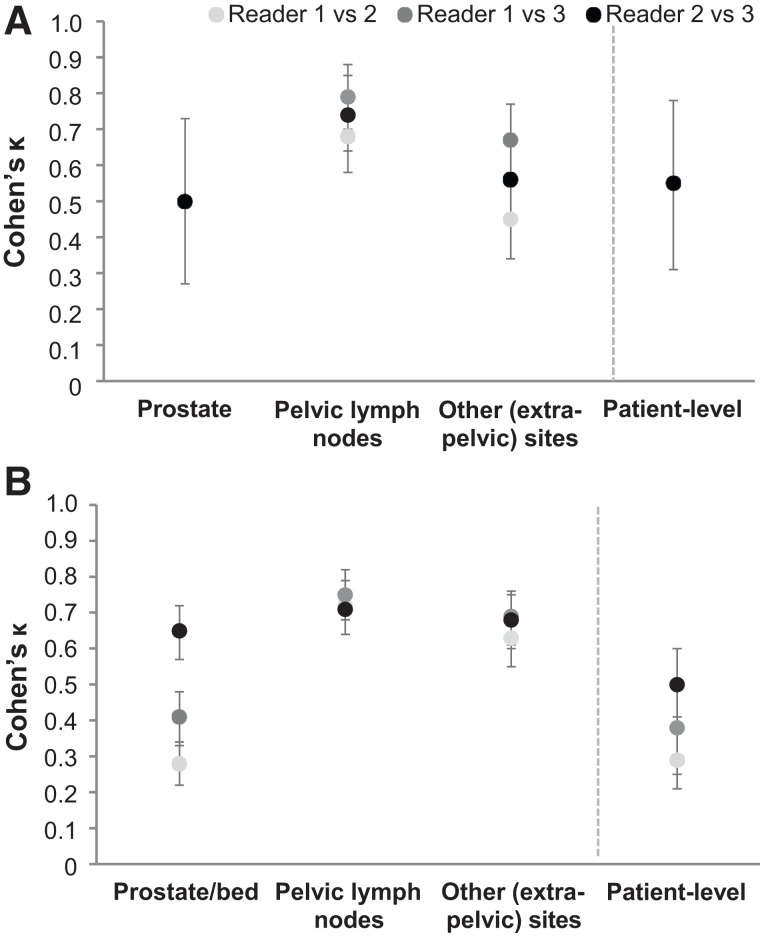
(A and B) Cohen κ for pairwise patient- and region-level interreader agreement of reads or images from 352 newly diagnosed (A) and 389 recurrent (B) PCa patients with evaluable ^18^F-flotufolastat PET/CT scan. Cohen κ was not estimable in prostate or at patient level for reader 1 vs. reader 2 or for reader 1 vs. reader 3 because of lack of negative prostate reads in newly diagnosed population.

Cohen κ for the pairwise interreader agreement is presented in Figure 2. Across both populations, Cohen κ for pairwise interreader agreement ranged from nonestimable to 0.55 at the patient level, from nonestimable to 0.65 in the prostate or prostate bed, from 0.68 to 0.79 in PLN, and from 0.45 to 0.69 in extrapelvic sites. Supplemental Table 1 provides the interreader agreement and Cohen κ for the various subcategories of extrapelvic regions (supplemental materials are available at http://jnm.snmjournals.org).

**FIGURE 3.**
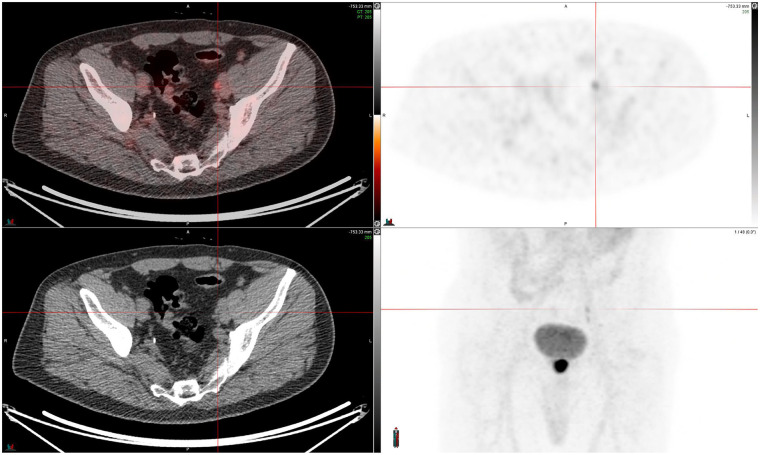
Discordant case in left external iliac lymph node. Fused PET/CT and ^18^F-flotufolastat PET (right) images (SUV, 0–10) from newly diagnosed high-risk PCa patient (PSA, 17.8 ng/mL) are shown. One of 3 masked readers identified metastasis in left external iliac lymph node, which other 2 readers did not read as positive. Lesion was later confirmed as true positive by histopathology, resulting in majority-read false-negative result.

Figure 3 presents a case study of a discordant read in an external iliac lymph node in a patient with newly diagnosed PCa.

As shown in Supplemental Tables 2 and 3, which present the patient-level interreader agreement stratified by PSA level, although the small numbers of patients in some PSA categories limit the conclusions that can be drawn, high levels of agreement were seen irrespective of PSA levels among newly diagnosed patients, and the recurrent data suggest a trend toward higher agreement in patients with a PSA level greater than 1 ng/mL than in patients with a PSA level less than 1 ng/mL.

Fleiss κ, which was used to assess reader agreement for all 3 readers across 5 regions in each patient, was 0.50 (95% CI, 0.46–0.53) in the newly diagnosed population and 0.41 (95% CI, 0.39–0.43) in the recurrent population.

### Intrareader Agreement

**FIGURE 4.**
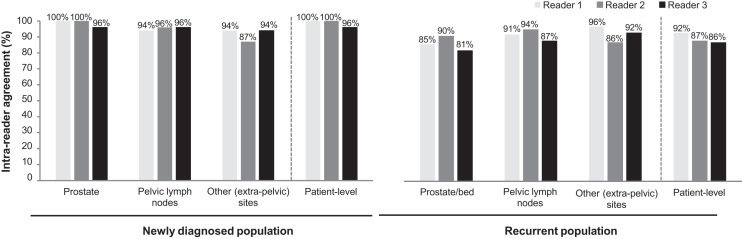
Patient- and region-level intrareader agreement for reread of ^18^F-flotufolastat PET/CT images from newly diagnosed (*n* = 70) or recurrent (*n* = 78) PCa patients.

In total, 70 scans from the LIGHTHOUSE study and 78 scans from the SPOTLIGHT study were reread at least 4 wk after the initial read to determine the intrareader agreement. Figure 4 presents the intrareader agreement between the 2 reads.

Across both studies, the patient-level intrareader agreement was 86% or better for each reader. On a regional basis, intrareader agreement was broadly high for each reader, with agreement highest in the prostate region for newly diagnosed patients and in extraprostatic regions in patients with recurrent PCa.

**FIGURE 5.**
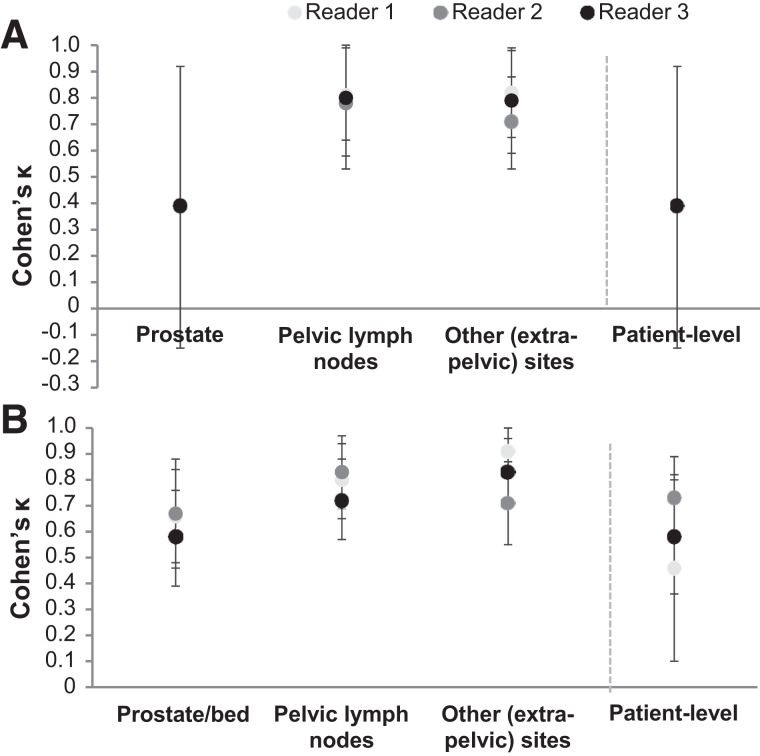
Cohen κ for patient- and region-level intrareader agreement for reread of ^18^F-flotufolastat PET/CT images from newly diagnosed (A) (*n* = 70) and recurrent (B) (*n* = 78) PCa patients. Cohen κ was not estimable in prostate or at patient level for readers 1 and 2 because of lack of negative prostate reads in newly diagnosed population.

Cohen κ for the intrareader agreement is shown in Figure 5. Cohen κ was higher for PLN and other (extrapelvic) regions than for the prostate or prostate bed. In scans from newly diagnosed patients, Cohen κ was estimable for the prostate for only 1 reader.

## DISCUSSION

The phase 3 LIGHTHOUSE and SPOTLIGHT studies show ^18^F-flotufolastat to have a clinically meaningful diagnostic performance in newly diagnosed and recurrent PCa (3*,*^4^). However, determining that results can be consistently and reliably interpreted is imperative for its clinical use. Here, we report data from these 2 trials to evaluate the inter- and intrareader agreement of ^18^F-flotufolastat PET/CT image interpretation.

Although some differences among the readers were noted, particularly in the prostate or prostate bed of patients with recurrent PCa, overall, the data demonstrate that naïve readers who had received specific ^18^F-flotufolastat reader-interpretation training were able to achieve good reproducibility when staging newly diagnosed or recurrent PCa.

In newly diagnosed patients, patient-level interreader agreement was 95% or better. As might be expected in this newly diagnosed treatment-naïve population, the region-level agreement was highest in the prostate. Reassuringly, however, there was also good agreement in extraprostatic regions among readers. Interreader agreement in PLN ranged from 92% to 95%. The corresponding Cohen κ was 0.68–0.79, marginally higher than that reported for ^68^Ga-PSMA-11 PET imaging (9). Reliably determining the presence of nodal and metastatic disease is particularly important in these patients given that such findings would be more likely to influence treatment decisions (1). We observed low or nonestimable κ coefficients for corresponding high agreement data in some analyses. This high-agreement–low-κ paradox is a consequence of the κ coefficient’s propensity to be affected by the prevalence of rare findings. For example, as the readers rarely reported the prostate region in newly diagnosed patients to be negative, inter- and intrareader κ coefficients for the prostate and patient level were affected despite high levels of agreement.

Although slightly lower levels of interreader agreement were observed in the recurrent population than in the newly diagnosed population, the patient-level interreader agreement for ^18^F-flotufolastat was still high at 75%–86% and was comparable with other PSMA PET agents. The CONDOR trial data show an interreader agreement of 75% among 3 readers of ^18^F-DCFPyL scans from 208 patients with recurrent PCa (10), whereas pairwise patient-level interreader agreement data for ^68^Ga-PSMA-11 PET imaging in 125 patients with metastatic castration-resistant PCa are reported as 82%–88% (11). Our interreader agreement data in the recurrent population were impacted by findings from reader 1, who read considerably more scans as positive in the prostate region than did the other masked readers (4) and the onsite readers (Supplemental Table 4). Reproducibility in extraprostatic regions was high and of a similar level to that seen in newly diagnosed patients.

Interpretation standards for strength of agreement based on κ coefficients have been proposed (12*,*^13^), and although these standards, particularly those by Landis and Koch (12), are widely applied to the interpretation of the κ coefficient, they are somewhat arbitrary and should be used with caution given the effects of prevalence and bias on the κ coefficient and the high-agreement–low-κ paradox discussed above (14). Landis’s and Koch’s scale ranks agreement as poor (κ < 0), slight (0.0 > κ < 0.20), fair (0.21 > κ < 0.40), moderate (0.41 > κ < 0.60), substantial (0.61 > κ < 0.80), or almost perfect (0.81 > κ < 1.0). Application of these interpretation scales here suggests that the Fleiss κ for agreement among all 3 readers, which were likely impacted by the prostate region reporting discussed above, shows moderate κ coefficients (12). Patient-level intrareader agreement was high throughout, again similar to that of other PSMA PET agents (11) but with κ coefficients that would broadly be considered moderate to substantial in the recurrent population and, where estimable, fair in the newly diagnosed population.

There are some limitations to the present analysis. First, as noted above, we observed a low κ coefficient for corresponding high-agreement data in some inter- and intrareader analyses across both populations, perhaps suggesting the unsuitability of the κ coefficient for these data. The κ coefficient can be unreliable for rare observations (such as negative ^18^F-flotufolastat PET/CT in the prostate), because of its propensity to be affected by the prevalence of the finding under consideration (15*,*^16^). This is perhaps demonstrated by the numerically lower κ coefficients (range, 0.29–0.5) for the patient-level pairwise interreader agreement in our recurrent population compared with the ^68^Ga-PSMA-11 PET data discussed above, which correspond with a κ coefficient of 0.54–0.67 despite similar levels of pairwise interreader agreement. Second, as is typical for trials of this type, scans were read by only a small number of readers, which means the impact of any outlier data such as the increased positive reads in a particular region by one of the readers is amplified. In a real-world environment, with access to patients’ clinical information, and with learning from follow-up of subjects, further increases in diagnostic accuracy and interreader agreement might be expected. Comparison of the reads between the onsite readers and the masked readers (Supplemental Table 4) perhaps exemplifies this and further highlights the outlier data in the prostate region in recurrent patients from reader 1. Among all cases in which the prostate was read negative by the unmasked onsite readers, positive reads by readers 2 and 3 were relatively uncommon at 3%–12%, whereas reader 1 read the prostate positive in 34% of these cases.

## CONCLUSION

The data here show ^18^F-flotufolastat PET/CT scans of patients with newly diagnosed and recurrent PCa can be reliably interpreted with a high degree of inter- and intrareader agreement. The high reproducibility of results observed here offers further support of the clinical utility of ^18^F-flotufolastat PET/CT imaging for patients with PCa.

## DISCLOSURE

Phillip Kuo is a consultant or speaker for Blue Earth Diagnostics, Chimerix, Eli Lilly, Fusion Pharma, GE Healthcare, Invicro, Novartis, Radionetics, and Telix Pharmaceuticals. He is a recipient of research grants from Blue Earth Diagnostics and GE Healthcare. Gary Ulaner has served as a speaker, received grant support, or served on a scientific advisory board for GE Healthcare, Lantheus, Nuclidium, and RayzeBio. Katherine Zukotynski has served on an advisory board for AAA/Novartis and as a consultant for Fusion Pharmaceuticals. Gregory Ravizzini is a recipient of research grants from GE Healthcare, Curium, ABX, Bayer, Novartis, Clarity, and Fusion. David Schuster has acted as a consultant for Global Medical Solutions Taiwan; Progenics Pharmaceuticals, Inc.; Heidelberg University; and DuChemBio Co. Ltd. He participates through the Emory Office of Sponsored Projects in full compliance with Emory University sponsored research and conflict-of-interest regulations in sponsored grants including those funded or partially funded by Blue Earth Diagnostics; Nihon MediPhysics Co., Ltd.; Telix Pharmaceuticals (U.S.) Inc.; Advanced Accelerator Applications; FUJIFILM Pharmaceuticals U.S.A., Inc; and Amgen Inc. He participates in educational initiatives with School of Breast Oncology and PrecisCa and provides medicolegal consulting vetted through Emory School of Medicine. No other potential conflict of interest relevant to this article was reported.

## Notes

KEY POINTS **QUESTION:** How reliably can ^18^F-flotufolastat scans of patients with newly diagnosed and recurrent PCa be interpreted? **PERTINENT FINDINGS:** ^18^F-flotufolastat scan interpretation by 3 masked readers in two phase 3 clinical trials show the pairwise interreader agreement to be 95% or higher in newly diagnosed patients and 75% or higher in patients with recurrent PCa. Intrareader agreement was 86% or higher in both populations. **IMPLICATIONS FOR PATIENT CARE:** As this newly approved PET/CT imaging agent moves into clinical use, the present study shows that it can be reliably interpreted to give consistent results across individual readers.
